# Human hair follicle transcriptome profiling: a minimally invasive tool to assess molecular adaptations upon low‐volume, high‐intensity interval training

**DOI:** 10.14814/phy2.13534

**Published:** 2017-12-07

**Authors:** Jing Zhang, Sarah J. Wallace, Maria Y. Shiu, Ingrid Smith, Shawn G. Rhind, Valerie S. Langlois

**Affiliations:** ^1^ Chemistry and Chemical Engineering Department Royal Military College of Canada Kingston ON Canada; ^2^ Defense Research and Development Canada Toronto Research Centre Toronto ON Canada; ^3^ Present address: Schulich School of Medicine and Dentistry Western University London ON N6A 5C1 Canada

**Keywords:** Endurance training, energy metabolism, miRNA, muscle contraction, RNA‐Seq

## Abstract

High‐intensity interval training (HIIT) has become a popular fitness training approach under both civilian and military settings. Consisting of brief and intense exercise intervals, HIIT requires less time commitment yet is able to produce the consistent targeted physical adaptations as conventional endurance training. To effectively characterize and monitor HIIT‐induced cellular and molecular responses, a highly accessible yet comprehensive biomarker discovery source is desirable. Both gene differential expression (DE) and gene set (GS) analyses were conducted using hair follicle transcriptome established from pre and postexercise subjects upon a 10‐day HIIT program by RNA‐Seq, Comparing between pre and posttraining groups, differentially expressed protein coding genes were identified. To interpret the functional significance of the DE results, a comprehensive GS analysis approach featuring multiple algorithms was used to enrich gene ontology (GO) terms and KEGG pathways. The GS analysis revealed enriched themes such as energy metabolism, cell proliferation/growth/survival, muscle adaptations, and cytokine–cytokine interaction, all of which have been previously proposed as HIIT responses. Moreover, related cell signaling pathways were also measured. Specifically, G‐protein‐mediated signal transduction, phosphatidylinositide 3‐kinases (PI3K) – protein kinase B (PKB) and Janus kinase (JAK) – Signal Transducer and Activator of Transcription (STAT) signaling cascades were over‐represented. Additionally, the RNA‐Seq analysis also identified several HIIT‐responsive microRNAs (miRNAs) that were involved in regulating hair follicle‐specific processes, such as *miR‐99a*. For the first time, this study demonstrated that both existing and new biomarkers like miRNA can be explored for HIIT using the transcriptomic responses exhibited by the hair follicle.

## Introduction

High‐intensity interval training (HIIT) involves brief repeated bouts of near maximal exercise (i.e., 80–100% max heart rate and/or power output) interspersed with short recovery periods and has been shown to elicit comparable health and performance benefits to those associated with conventional moderate‐intensity continuous training (MICT) (Gibala et al. 2012; Boyne et al. 2013). Moreover, with its effectiveness in improving aerobic capacity and reduction in time‐commitment, HIIT has become a highly popular exercise training modality, with its utility ranging from everyday fitness, rehabilitation programs, to military physical training protocols (Boyne et al. 2013; Gillen and Gibala 2014).

Understanding the spectrum of physiological and biomolecular remodeling underlying the HIIT‐responsive cellular processes has been largely conducted using invasive methods, including muscle biopsies, peripheral blood, or other tissue samples in either human or rodent models. While critical to characterizing HIIT‐derived adaptations directly from the tissues of interest, a minimally invasive yet sensitive biomarker discovery source is valuable for a comprehensive assessment of HIIT responses in a timely fashion. Hair follicle controls hair production and development through hair cycle, which requires close cross‐talk between cellular processes and between different cell types, including follicular stem cells, and fully differentiated cells like keratinocytes (Stenn et al. 1994; Paus and Cotsarelis 1999; Stenn and Paus 2001; Botchkarev and Paus 2003). As such, hair follicle represents a versatile system that could reflect physiological adaptations through numerous cellular and molecular processes, thereby providing sources for biomarker discovery. Indeed, mammalian hair follicle was already proposed as a biomarker discovery system for various diseases and health conditions (Kim et al. 2006; Paus et al. 2006; Choi et al. 2011; Roberts et al. 2014; Maekawa et al. 2015). It has also been suggested as a potential minimally‐invasive model to study global changes in gene expression with various modes of physical activity (Takahashi et al. 2017). Moreover, we have previously demonstrated the viability of using rat whisker hair follicle for transcriptomic biomarker discovery in blast‐induced mild traumatic brain injury (Zhang et al. 2014). Therefore, existing and new transcriptomic responses might be present in the hair follicles for HIIT, making the system ideal for evaluating effectiveness of the training regime.

A wide range of HIIT‐associated cellular and molecular processes that govern the corresponding physiological and biochemical adaptations has been discovered. For example, the brief yet intense muscular contraction occurring with the training bouts induces alterations to the cell signaling pathways controlling energy metabolism and cell proliferation/growth, such as AMP‐activated protein kinase (AMPK) and phosphatidylinositide 3‐kinases (PI3K) – protein kinase B (PKB) pathways (Atherton et al. 2005; Gibala 2009; Lu et al. 2015). Additionally, a recent study demonstrated HIIT stimulates muscular adaptation by altering fat and carbohydrate metabolic pathways in skeletal muscle with elevated mitochondrial function and changes in regulatory steps of metabolic and signaling pathways (Perry et al. 2008). Improvement of mitochondrial oxidative phosphorylation capacities were observed upon completion of HIIT, resulting from enhanced expression and activity of the mitochondrial enzymes, including cytochrome c oxidase (COX) and citrate synthase (CS) (Little et al. 2010; Ramos‐Filho et al. 2015). As such, HIIT activates stress tolerance and cellular survival mechanisms, including antioxidant defenses and apoptosis, which can be regulated by mitogen‐activated protein kinase (MAPK) signal transduction cascades and peroxisome proliferator‐activated receptor (PPAR) pathway (Gibala et al. 2012; Lu et al. 2015). In addition to muscle adaptations, increased insulin sensitivity of the liver and adipose tissue have been proposed as part of the HIIT responses, which were also related to AMPK and PI3K‐PKB pathways (Marcinko et al. 2015). Furthermore, immuno‐inflammatory responses (IR) have also been closely related to HIIT (Zwetsloot et al. 2014; Cullen et al. 2016; Elmer et al. 2016; Kaspar et al. 2016). Taken together, it is possible to use the aforementioned molecular biomarkers in combination with specific metabolic parameters (such as heart rate, respiratory gas exchange, mitochondrial function, and energy metabolism) to monitor and study HIIT‐induced effects (Gibala et al. 2012; Marcinko et al. 2015).

This study examines the utility of human hair follicle in exploring the transcriptomic responses upon a 2‐week HIIT regime. By conducting RNA‐Seq using total RNA library, we were able to explore transcripts from both coding and microRNA (or miRNA) features. Based on the RNA‐Seq gene expression profiling, differentially expressed genes were identified upon HIIT. Gene set (GS) analyses were then carried out to depict the potential biological and physiological consequences to the gene differential expression (DE) results. This is the first study to examine human hair follicle as a minimally invasive transcriptome profiling tool to study adaptations associated with short‐term HIIT.

## Materials and Methods

### Participants and ethical approval

Four healthy recreationally active men (22–32 years of age; body mass index 24.3 ± 3.3 kg m^–2^, mean ± standard deviation) were recruited from Canadian Armed Forces (CAF) personnel through the Area Support Unit Toronto, including the Canadian Forces Environmental Medical Establishment (CFEME), and Defence Research and Development Canada (DRDC), Toronto Research Centre. The subjects completed a Physical Activity Readiness Questionnaire, a standard medical exam and provided their written informed consent prior to their participation. The DRDC Human Research Ethics Committee and University of Toronto Research Ethics Board approved the study.

### Pretraining procedures

Preliminary testing was completed 1 week before the first trial. All participants completed a standardized maximal graded ramp test to exhaustion on an electronically braked cycle ergometer (Velotron Dynafit Pro, RacerMate Inc., Seattle, WA) to determine whole‐body peak oxygen uptake (V̇O_2peak_ test). Following a 1 min warm‐up at 50 W, the workload was increased at a rate of 25 W every minute until the subject reached volitional exhaustion or the cadence decreased below 60 rpm. Participants were fitted with an electronic heart rate monitor (Polar WearLink+, Polar Electro Canada, Lachine, QC) for determination of resting heart rate and to measure heart rate during the exercise test. Expired respiratory gases were measured continuously by an automated breath‐by‐breath metabolic system (MOXUS Modular Metabolic System, AEI Technologies, Inc., Pittsburgh, PA) and the V̇O_2peak_ value was determined by the maximum oxygen uptake over a 15 sec period. The mean V̇O_2peak_ and heart rate of the subjects were 43.25 ± 6.4 ml kg^−1^ min^−1^ (mean ± standard deviation) and 178 ± 14 bpm (mean ± standard deviation), respectively.

### Training

All training was performed on the same cycle ergometer as that used for baseline testing. A minimum of 48 h and no more than 10 days following the initial V̇O_2peak_ test, participants began the 2‐week HIIT regimen every other day, with three sessions a week, completing six HIIT sessions in 2 weeks. The protocol comprised a 3‐min warm up at 50 W followed by eight repeated 60‐sec intervals of cycling during session 1 and 2, 10 intervals during session 3 and 4, and 12 intervals during session 5 and 6 at a workload corresponding to 100% of each participant's predetermined V̇O_2peak_, interspersed with 75‐sec active recovery period at 50 W. Participants completed the protocol with a 3‐min cool‐down at 50 W. Participants maintained the 60 rpm cadence throughout the sessions.

### Posttraining procedures

Minimum of 24 h, but no more than 48 h upon completion of the last HIIT session, participants performed a posttraining V̇O_2peak_ test. The V̇O_2peak_ test protocol was identical to that of the pretraining V̇O_2peak_ test.

### Sample collection and RNA extraction

Approximately 10 hair follicles at the vertex area of the scalp were collected per participant before (pre‐HIIT) and upon completion (post‐HIIT) of a 2‐week HIIT regimen. The sampled hair follicles were preserved by RNA later solution (Qiagen, ON, Canada) for future batch analysis.

Total RNA was extracted for both RNA‐Seq and quantitative PCR (qPCR) analyses. An RNeasy Micro Kit (Qiagen) was used for hair follicle RNA extraction. Briefly, samples were homogenized using a sonicator (ThermoFisher, ON, Canada) in a homogenization buffer prior to the extraction and purification steps with the HiBind RNA Spin Columns. The kit provided both homogenization buffer and the spin columns. The concentration and purity of the RNA extracts were determined using a NanoDrop spectrophotometer (ThermoFisher); and the integrity was determined as RNA integrity number (RIN) on a Bioanalyzer (Agilent Technologies, ON, Canada). RNA samples with the minimum RIN of seven were used for further analyses.

### RNA‐Seq

Total RNA libraries were prepared using Illumina TruSeq total RNA library prep kit (Illumina, BC, Canada) with Ribo‐Zero ribosomal RNA (rRNA) depletion kit (Illumina) following manufacturer's instruction. All resulting libraries were checked for size and concentration using Bioanalyzer (Agilent Technologies) and Qubit (Thermo Fisher Scientific, ON, Canada), respectively. Sequencing runs were performed on a MiSeq instrument (Illumina) with the pair‐ended sequencing kit (Illumina). To achieve the maximum sequencing depth available, individual runs were carried out for each sample instead of multiplexing. All raw data were deposited to short reads archive (SRA) database as BioProject (https://www.ncbi.nlm.nih.gov/bioproject/, BioProject ID: PRJNA396030).

### RNA‐Seq data preprocessing and gene differential expression (DE) analysis

The raw sequencing reads from all the samples were aligned to the human genome (release version hg19) using TopHat (Trapnell et al. 2009) through Illumina's BaseSpace cloud service (Illumina). Prior to read counting, software samtools (Li et al. 2009) was used to sort the bam files resulted from the sequence alignment by the name of the genome features. Reads were then counted for all the genomic features using HTseq‐count (Anders et al. 2015) with the annotation file corresponding to the reference genome version. With the rest settings being default, feature type, counting mode and stranded were set to “gene,” “intersection‐nonempty,” and “reverse,” respectively. The raw read counts were then filtered and normalized using the voom method (Law et al. 2014) through the R package limma (Ritchie et al. 2015).

The R package limma (Ritchie et al. 2015) was also used for gene differential expression (DE) analysis. Generally, statistical analysis was based on linear model fitting with empirical Bayesian test. The empirical Bayesian test p values were corrected using FDR (false discovery rate) with an alpha value set to 0.05. According to the scope of this study, genome features under the categories of protein coding and miRNA (microRNA) were extracted for further analysis.

### Quantitative PCR

qPCR was used to confirm the DE results from RNA‐Seq analysis. A subset of the genes that showed DE signals were used for qPCR validation (see Table 1 for details), with *tubulin alpha 1a* (*tuba1a*) and *let‐7a* used as the housekeeping genes, which exhibited a consistent expression level in both groups. All primers were designed using the Primer‐BLAST tool from NCBI (http://www.ncbi.nlm.nih.gov/tools/primer-blast/) and their sequence can be found in Table 1. For mRNAs, cDNA was synthesized from the high‐quality total RNA using a QuantiTect Reverse Transcription Kit (Qiagen).

**Table 1:** Primer sequences for the genes analyzed using qPCR with optimized conditions including annealing temperature and primer concentration. Key functions are also listed for each gene. F: forward; R: reverse

Gene	Main function	Sequence	Annealing temperature (°C)	Primer concentration (nmol L^−1^)
*krt78*	Keratin protein	F: 5′‐TGGTCCTCAAGAAGGATGTGG‐3′	60	350
		R: 5′‐GATGCTGCTGAAGTCCAGGT‐3′		350
*slc2a5*	Glucose transport	F: 5′‐TATCGGATCCCTCCTGGTCG‐3′	60	350
		R: 5′‐CACGTTGGAAGATACACCTGC‐3′		350
*rilpl2*	Lysosome morphology	F: 5′‐GTGCTACAAGAGTGGCCTGAT‐3′	60	300
		R: 5′‐AGCCTTACTTGTGGCCTTGG‐3′		300
*gpr128*	Cell signaling	F: 5′‐CTGGAAACCCTGGAAAAGCA‐3′	60	300
		R: 5′‐ATGGCAACTTTCTTTGCCTCA‐3′		300
*Tuba1a*	Housekeeping gene	F: 5′‐GAAGCAGCAACCATGCGTGA‐3′	60	300
		R: 5′‐ATCTCCTCCCCCAATGGTCTT‐3′		300
*let‐7a*	Housekeeping gene	F: 5′‐AGCAGTGAGGTAGTAGGT‐3′	60	250
		R: 5′‐ CCAGTTTTTTTTTTTTTTTAACTATACA‐3′		
*leg‐7g*	Apoptosis	F: 5′‐GCAGTGAGGTAGTAGTTTGTA‐3′	60	250
		R: 5′‐GTCCAGTTTTTTTTTTTTTTTAACTG‐3′		
*miR‐99a*	Keratinocyte differentiation	F: 5′‐AGAACCCGTAGATCCGA‐3′	60	250
		R: 5′‐CAGTTTTTTTTTTTTTTTCACAAGA‐3′		
RT primer	N/A	5′‐CAGGTCCAGTTTTTTTTTTTTTTTVN– 3′		

For miRNA amplification, cDNA was synthesized with the additional poly‐adynlation step using a Poly(A) Polymerase Tailing Kit (Epicentre Technologies, IL). Briefly, 200 ng high‐quality total RNA was the used in the poly(A) tailing reaction for each sample with 1 U of poly(A) polymerase associated with the kit. The reactions were carried out at 37°C for 30 min. The resulting RNA samples were subjected to cDNA synthesis using an RT primer specifically targeting poly‐adenylated RNA.

Prior to the quantification runs, all primer sets were tested and optimized using cDNA samples pooled from both groups for an amplification efficiency between 90 and 110% and a linear regression coefficient between 0.9 and 1.0 for standard curves. A CFX thermocycler (BioRad, Canada) was used for qPCR amplification with GoTaq qPCR master mix (Promega, WI). The amplification process was programmed as following: 95°C for 3 min, 40 cycles of 95°C for 15 sec and 58–62°C for 1 min. A dissociation curve was generated over the temperature range of 60–95°C immediately after each qPCR reaction, to confirm single product amplification. Optimized qPCR conditions for each gene can also be viewed in Table 1. Raw data acquisition was carried out using the CFX manager (Biorad, Canada) software. Student's *t*‐test was used for statistical analysis for the qPCR amplification using the JMP SAS 9.0 software (SAS, NC). Changes with a *P* value from Student's *t*‐test <0.05 were considered statistically significant.

### Gene set (GS) analysis

Gene ontology (GO) term and KEGG pathways (Ashburner et al. 2000; Luo and Brouwer 2013; Maekawa et al. 2015) were used for GS analysis to further evaluate the biological and functional significance of the DE signals discovered from RNA‐Seq experiment. For GO term analysis, biological processes (BP) and molecular functions (MF) sets were assessed. In addition to GO term, KEGG pathways provides both more functional insights of the DE results and a detailed visual representation of the HIIT‐responsive pathways and processes. All GS enrichment tests were carried out using the R package piano (Väremo et al. 2013).

To take the overall gene expression profile into account, the gene level statistics (i.e., fold change/*t*‐statistics and *P* value) for the whole protein coding gene list were used for the GS analysis. Firstly, the nondirectional GS test uses the absolute value of gene level fold change or *t*‐statistics, and thus reveals the pathways that might be regulated by HIIT based on the overall DE results. Moreover, adding the directionality information present in the gene level statistics (i.e., up and down regulation) to the GS analysis provides a more detailed functional analysis to the regulations on enriched pathways. Directional GS analyses feature distinct directional enrichment and mixed directional enrichment. For the distinct directional test, with the opposite DE signals cancelling out, significantly regulated gene sets can only be in one direction. On the other hand, enriched pathways can be regulated in both directions in the mixed directional test if the pathway contains subsets of genes show either one direction from the DE results. For directional methods, GS *P* values are calculated for both up and down regulations.

This study utilized multiple GS enrichment algorithms through a consensus score to minimize method bias. A spectrum of GS tests was used, including both parametric (i.e., Fisher's combined probability, Stouffer's test, Reporter features test, and parametric analysis of gene set enrichment [PAGE] test) (Fisher 1932; Stouffer et al. 1949; Kim and Volsky 2005; Patil and Nielsen 2005), nonparametric methods (i.e., Tail strength test, Wilcoxon rank‐sum test, and gene set enrichment analysis [GSEA] test) (Smyth 2005; Subramanian et al. 2005; Taylor and Tibshirani 2006), as well as a “maxmean” method developed by Efron and Tibshirani (2007). For all eight enrichment tests, null distribution was generated by a gene sampling permutation (1000 times) approach. All GS level *P* values were adjusted by FDR with an alpha value set at 0.05. All GO terms and KEGG pathways tested were ranked for each enrichment test, with the median of the ranks used as the consensus score of the gene sets.

The overall results of the GS analysis were visualized in quadrant scatter plots and boxplots. For quadrant scatter plots, the consensus scores were plotted against median GS *P* value for the gene sets. Boxplots were used to show the top ranked gene sets (consensus score cut‐off: 15). Selected key pathways were also graphically represented with the corresponding DE results masked onto the figure objects. All plots were generated using the R packages pathview, reshape2, and ggplot2 (Wickham 2007, 2009; Luo and Brouwer 2013), as part of the data analysis pipeline specifically developed for this study.

## Results and Discussion

The advantage of HIIT resides in its relatively low time commitment and equal, if not superior, effect to the conventional endurance training, such as MICT (Little et al. 2010). Serving as the foundation of the physiological adaptations, molecular biomarkers from the HIIT‐responsive biological and cellular events not only provide additional layers of measurable metrics to evaluate training results, but also provide a tool for a comprehensive characterization for the HIIT‐derived adaptations. Moreover, a wide range of HIIT‐associated biological and cellular events house various types of biomarkers, such as protein factors and RNA transcripts derived from the corresponding genes. Conventional approaches of assessing HIIT‐responsive biomarkers largely include direct examination of various tissues of interest, such as blood and muscle biopsy. Additionally, lab rodent models have been frequently used to assess the organs that typically face challenging accessibility in human subjects upon HIIT, such as the liver (Marcinko et al. 2015). A minimally invasive transcriptomic biomarker system enables faster HIIT response evaluation and characterization, with a possibility of extending our understanding on the training scheme. Given the prior knowledge of being used as a biomarker discovery system (Roberts et al. 2014; Zhang et al. 2014; Maekawa et al. 2015), hair follicles were used to explore the HIIT‐responsive transcriptomic biomarkers.

**Figure 1:**
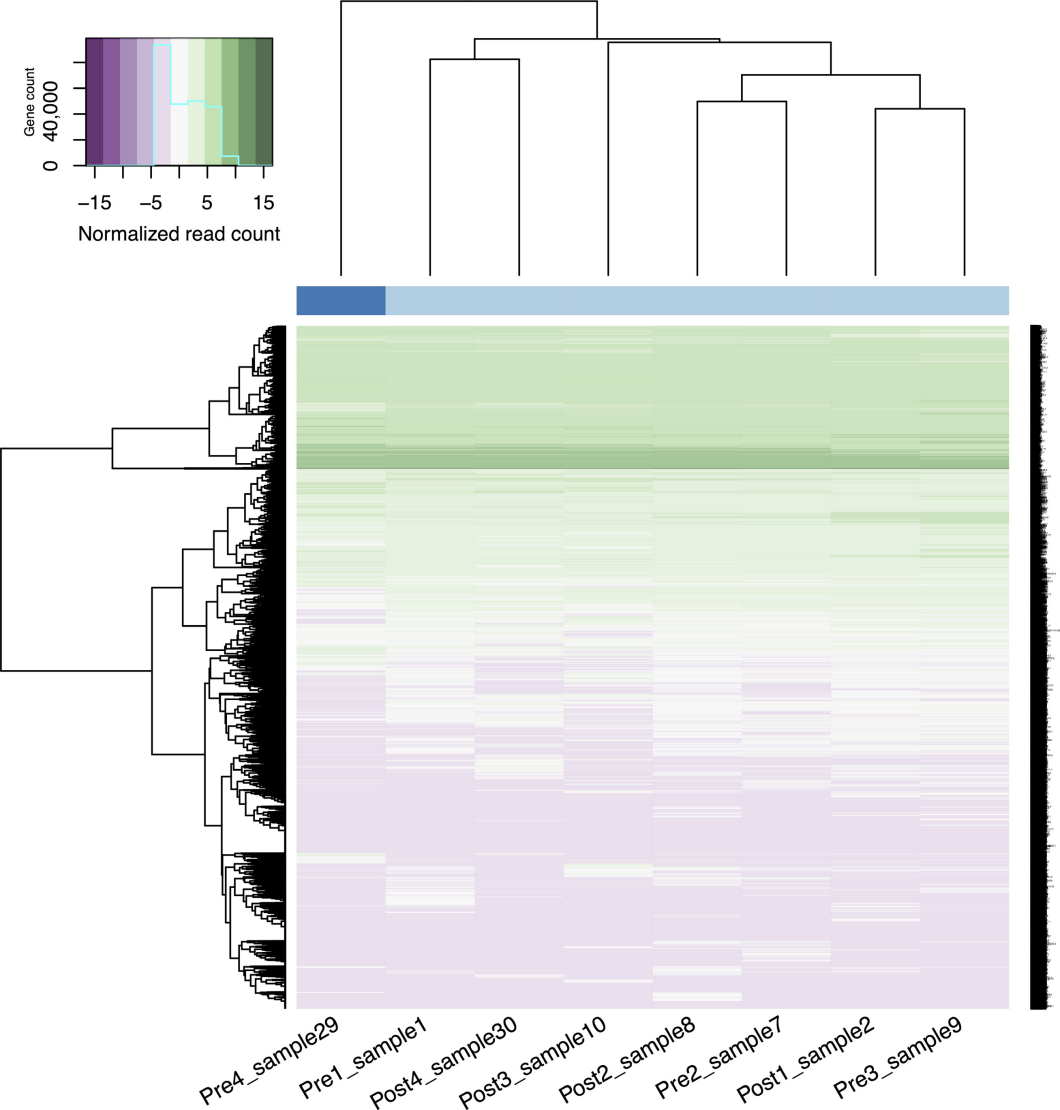
Heat map showing unsupervised hierarchical clustering analysis on the identified genomic features from pre‐ and post‐HIIT subjects.

**Figure 2:**
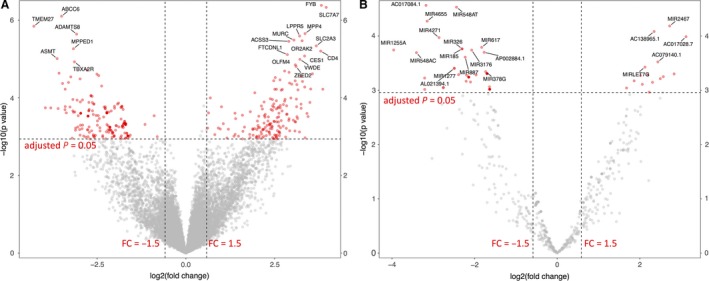
Volcano distribution for the differential expression analysis results for protein coding gene and miRNA expressions comparing pre‐ and post‐HIIT. Log_2_ FC (fold change) and −log_10_ *P* values are plotted. Red represents statistically significant changes >1.5‐fold. Target names are marked for the top 20 differential expressions. (A) protein coding genes; (B) miRNA.

The RNA‐Seq approach featured in this study was designed to provide a snapshot of pre‐ and post‐HIIT scalp hair follicle transcriptomes, providing a first look at the transcriptomic landscape under such conditions in hair follicles. Sequencing reads alignment revealed transcripts from both gene coding and noncoding genomic features, confirming that the scalp hair follicles indeed contain a rich amount of transcriptomic information. Based on the gene expression profile presented by the normalized read counts, the hierarchical clustering analysis exhibited great variance both within‐ and between‐individuals for pre‐ and post‐HIIT (Fig. 1). The clustering analysis also grouped genes with similar expression levels together, revealing both low and high abundance gene transcripts. Despite the high level of variance between subjects pre‐ and post‐HIIT, a comparison of expression level between the two groups discovered 1463 genomic features that were differentially expressed, of which 383 were protein coding genes. Volcano distribution was used to visualize DE results for the protein coding genes (Fig. 2A), showing 378 DE genes with a fold change equal to or greater than 1.5. Examples included *facilitated glucose/fructose transporter* (also known as *solute carrier family 2 member 5*, or *slc2a5*), *keratin 78* (*krt78*), and *G‐protein‐coupled receptor 128* (*gpr128*). It is worth noting that the RNAseq analysis also exhibited 66 differentially expressed miRNAs, even without small RNA enrichment steps during the library preparation procedure. As miRNAs regulate their mRNA targets via direct interaction (Chen and Rajewsky 2007), the miRNAs identified in this study might be part of the active miRNA:mRNA interaction complexes. As such, we found that hair follicles could indeed be used to reflect and explore the adjustment on gene expression upon HIIT. The complete DE results for protein coding genes can be view in Table S1 in supplementary materials.

To verify the RNA‐Seq DE results, qPCR was conducted on a selection of both DE protein coding genes and miRNAs. A summary of the results can be seen in Table 2. As shown in the table, all protein coding gene and miRNA targets showed a similar trend change pattern in the expression level between pre‐ and post‐HIIT in qPCR and RNA‐Seq analysis. Overall, all genes tested showed a matching direction in change, whereas only two targets exhibited discrepancy in p values between the two techniques. Accordingly, the RNA‐Seq DE results showed a high level of consistency with qPCR, suggesting the results from the former were valid. Further details for the top 20 DE genes marked on Figure 1 can be viewed in Table 3.

**Table 2:** The qPCR validation of the RNAseq results, with columns including gene name, exposure conditions, fold change *P*‐values, and correlation between the two methods

	RNAseq		qPCR		
Gene	Fold change	FDR adjusted* P‐*value	Fold change	*P*‐value	Validated (Y or N)
*tuba1a*	0.92	0.46	0.98	0.90	Y
*krt78*	4.33	0.045	7.97	0.035	Y
*slc2a5*	3.94	0.048	1.69	0.26	N
*rilpl2*	10.8	0.035	2.02	0.044	Y
*gpr128*	11.8	0.018	2.30	0.013	Y
*let‐7a*	0.28	0.65	1.07	0.86	Y
*let‐7g*	4.36	0.038	1.82	0.34	N
*mir‐99a*	0.15	0.045	0.51	0.045	Y

**Table 3:** Full gene name and the main function of the top 20 differentially expressed genes

Gene name	Full name	Main function (databases: NCBI, UniProt)
*tmem27*	*Transmembrane protein 27*	Trafficking amino acid transporter
*msln*	*Mesothelin*	Cell adhesion
*slc7a7*	*Solute carrier family 7 member 7*	The light subunit of a cationic amino acid transporter
*c11orf82*	*DNA damage‐induced apoptosis suppressor*	Potential antiapoptotic protein
*fyb*	*FYN‐binding protein*	T‐cell receptor signaling regulator
*tex37*	*Testis expressed 37*	Potential regulator for male spermatogenesis
*cd4*	*cd4 molecule*	T‐cell activation initiation, or potential central nervous system mediator to neuronal damage or disease
*slc2a3*	*Solute carrier family 2 member 3*	Glucose transporter
*asmt*	*Acetylserotonin O‐methyltransferase*	Regulator of melatonin synthesis
*kcna3*	*Potassium voltage‐gated channel subfamily A member 3*	Potassium channel component
*ac079602.1*	N/A	N/A
*gpr128*	Adhesion G‐protein‐coupled receptor G7	G‐protein‐coupled receptor
*cdhr4*	*Cadherin‐related family member 4*	Calcium‐dependent cell adhesion protein
*abcc6*	*ATP‐binding cassette subfamily C member 6*	Member of an extra and intracellular membranes molecule transporter
*mttp*	*Microsomal triglyceride transfer protein*	Subunit of a heterodimeric microsomal triglyceride transfer protein
*tekt3*	*Tektin 3*	Potential roles in cytoskeleton
*rilpl2*	*Rab interacting lysosomal protein like 2*	Cellular protein transport
*dmrta1*	*DMRT like family A1*	Transcription regulation
*actn2*	*Actinin alpha 2*	Part of cytoskeleton
*tmprss12*	*Transmembrane protease, serine 12*	Protease

**Figure 3:**
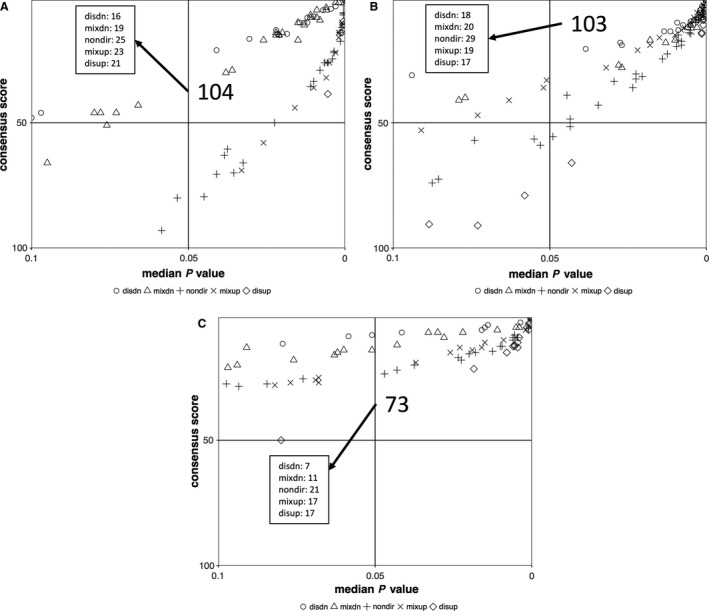
Quadrant scatter plots showing results of gene set (GS) analysis from both nondirectional and directional comparisons (five *P* value classes), using both gene ontology term (A, B) and KEGG pathway (C) gene sets. The upper right quadrant includes gene sets with a median GS *P* value <0.05 and were considered top ranked (i.e., a consensus score equal to or <50). The numbers indicate the quantity of enriched gene sets, both for total and individual *P* classes. Figure legend title explanation: disdn – distinct down upregulation, mixdn – mixed downregulation, nondir – nondirectional, mixup – mixed upregulation, disup – distinct upregulation.

**Figure 4:**
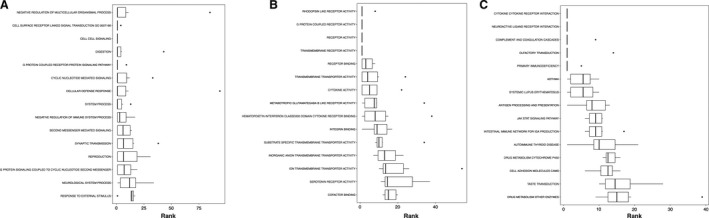
Boxplots showing the top ranked gene sets (i.e., with a consensus score equal to or small than 15) according to the consensus scores from all eight enrichment algorithms number nondirectional p class, (A) gene ontology term biological processes enrichment, (B) GP term molecular functions enrichment, (C) KEGG pathway.

**Figure 5:**
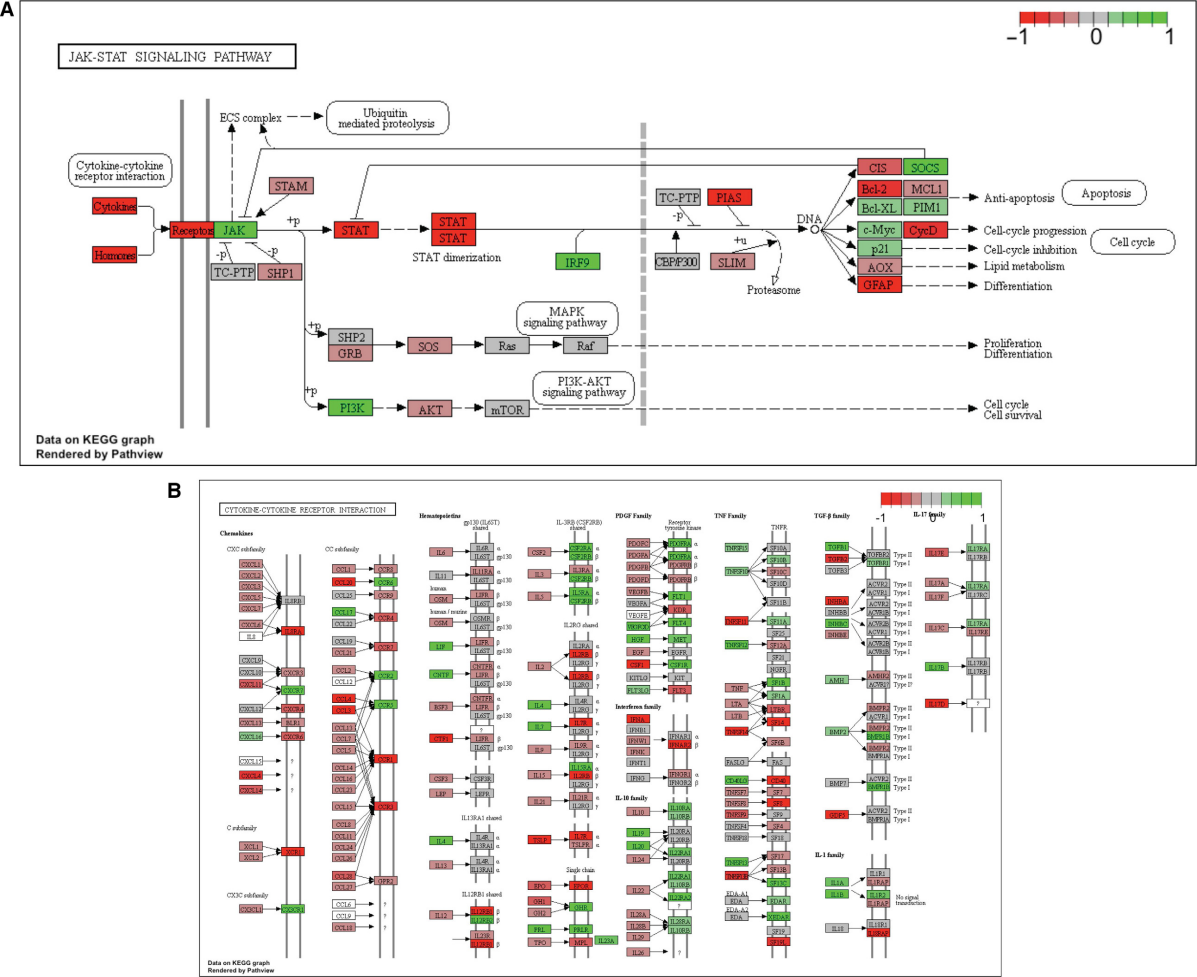
Graphical visualization of the key KEGG pathways that were considered distinctly downregulated: (A) JAK‐STAT signaling pathway, and (B) Cytokine‐cytokine receptor interaction. The differential expression analysis results for individual genes were shown through color masks on the boxes sanding for the corresponding genes, with a color gradient of red to green representing decreased to increased expression levels.

**Figure 6:**
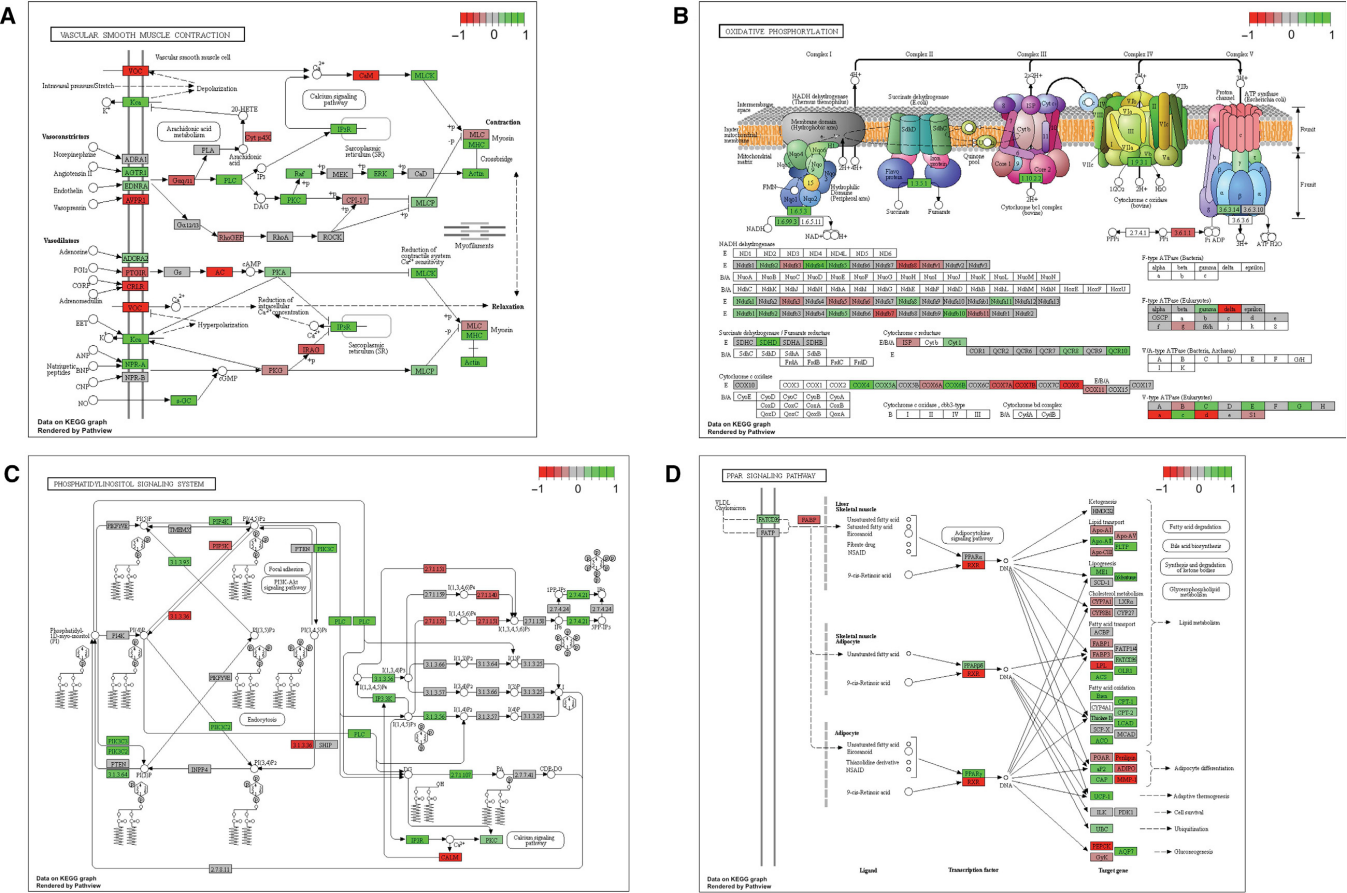
Graphical visualization of the key KEGG pathways that were considered distinctly upregulated, with (A) vascular smooth muscle contraction, (B) oxidative phosphorylation, (C) phosphatidylinositol signaling system, and (D) peroxisome proliferator‐activated receptor signaling pathway. Other information as Figure 5.

**Figure 7:**
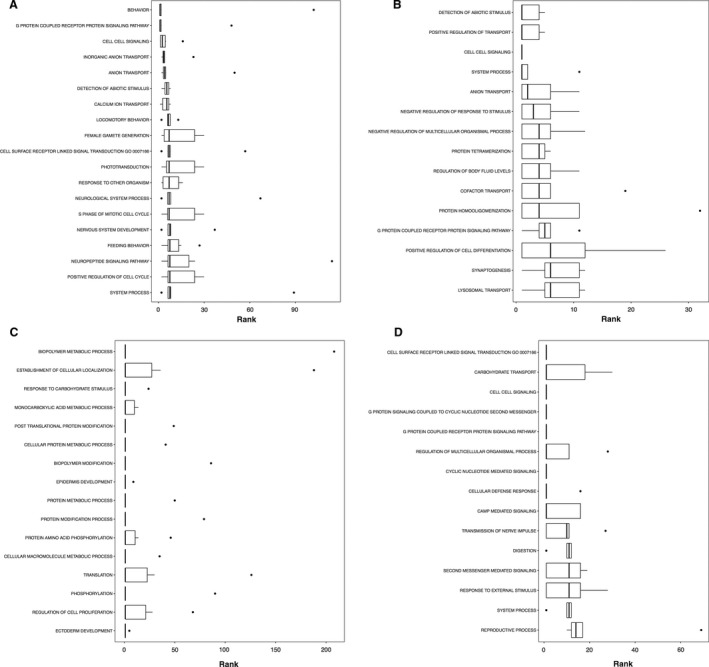
Boxplots showing the top ranked gene sets (i.e., with a consensus score equal to or small than 15) according to the consensus scores from all eight enrichment algorithms for gene ontology term biological processes enrichment: (A) distinct downregulations, (B) mixed downregulations, (C) distinct upregulations, and (D) mixed upregulations.

**Figure 8:**
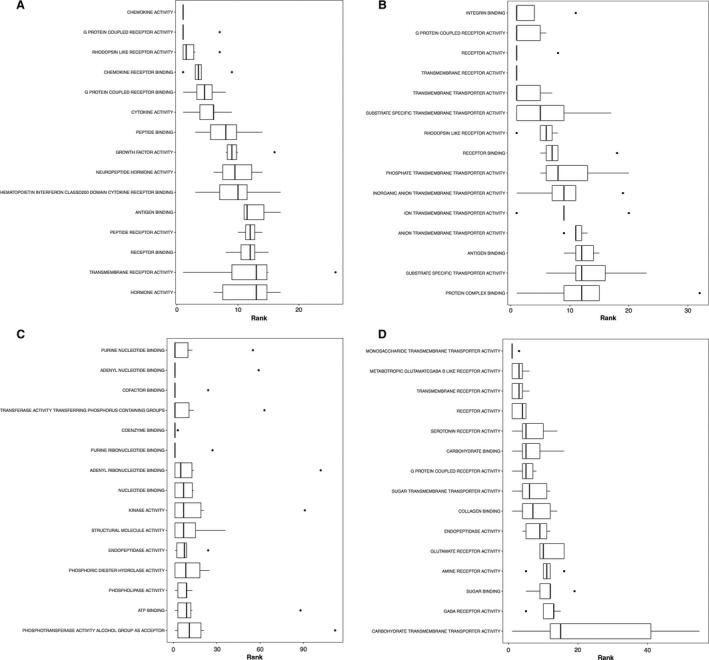
Boxplots showing the top ranked gene sets (i.e., with a consensus score equal to or small than 15) according to the consensus scores from all eight enrichment algorithms for gene ontology term molecular functions enrichment. Other information as Figure 7.

**Figure 9:**
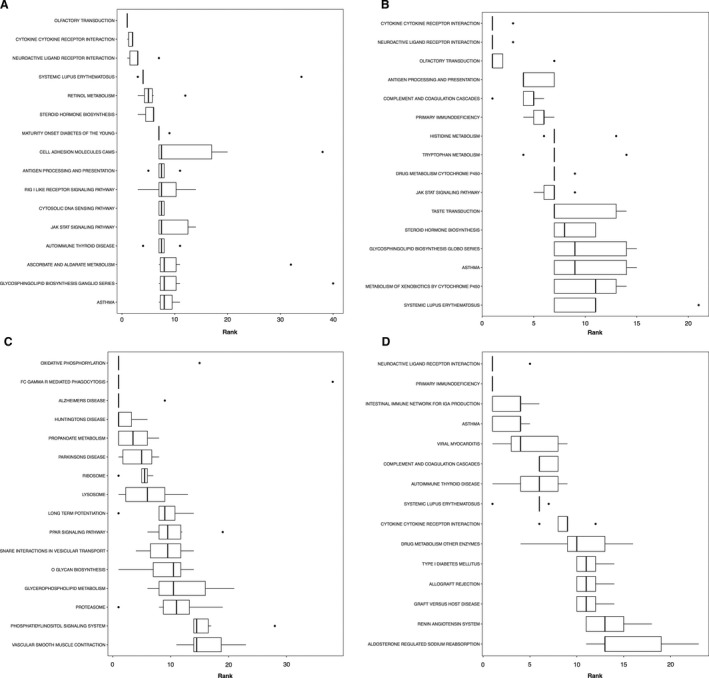
Boxplots showing the top ranked gene sets (i.e., with a consensus score equal to or small than 15) according to the consensus scores from all eight enrichment algorithms for KEGG pathway enrichment. Other information as Figure 7.

One crucial strategy to test the potential of scalp hair follicles serving as a biomarker discovery system for HIIT is to examine their capability of exhibiting well‐known HIIT molecular adaptations. As such, extensive GS analysis was conducted on the protein coding gene expression profiles from the DE analysis (Figs. 3, 4, 5, 6, 7, 8, 9). Overall, the DE results were able to enrich a myriad of GO terms and KEGG between pre and posttraining, suggesting hair follicle transcriptomes to be HIIT responsive. The results also showed both up and downregulated pathways, indicating a high versatility of the tissue. With our previous findings (Zhang et al. 2014), it appears that the mammalian hair follicle transcriptome is sensitive to different types of stressors. This emphasizes the potential of utilizing hair follicle analysis as a tool to study gene expression under remote or austere environments, or monitor health and performance in specialized populations including deployed military forces, which has been an active focus on military‐related research (Hoyt et al. 2002; Reifman et al. 2002).

Mediating various cellular and molecular processes, and signal transduction regulations is an integral part of the HIIT responses. Our GS analysis confirmed this was the case in hair follicles with enriched gene sets associated with signaling pathways and related cellular functions. First of all, nondirectional GO BP term enrichment showed overrepresentation of the terms such as Cell‐cell signaling, Cyclic nucleotide‐mediated signaling, and Second messenger‐mediated signaling. Consistently, a range of key molecular functions that contribute to signal transduction were also enriched, including G‐protein‐coupled receptor activity and Ion transmembrane transporter activity. Additionally, the directional enrichment of the gene sets further characterized HIIT‐responsive cell signaling. Specifically, BP term enrichment showed that both G‐protein‐coupled receptor (GPCR) activity and G‐protein‐coupled binding pathways were distinctly downregulated, meaning the overall effect of the DE signals contributed to the potential supressed gene expression involved in GPCR signaling cascades. However, for a selection of other related GO terms, a considerable amount of upregulated DE signals was included among them and drove a positive directionality in the mixed directional enrichment analysis, as seen for the BP term G‐protein‐coupled receptor activity, and cAMP‐mediated signaling. The qPCR results confirmed such assessment with the upregulated *gpr128* expression comparing between the pre‐ and post‐HIIT groups. These results are consistent with the reported critical and complex roles of GPCR signaling cascades in skeletal muscle regulations (Berdeaux and Stewart 2012; Garcia‐Guerra et al. 2014). Furthermore, several distinctly upregulated gene sets directly connected to kinase‐based cell signal transduction cascades were revealed in both GO term and KEGG pathway analyses. For GO BP terms, biological processes such as posttranslation modifications, protein modification process, protein amino acid phosphorylation, and phosphorylation were significantly upregulated; and the upregulation of molecular functions, including Kinase activity and ATP binding strongly correlated with the results from BP term enrichment. These results further confirmed the involvement of GPCR‐dependent signaling as it is also heavily dependent on phosphorylation events (Gurevich et al. 2012). More importantly, the kinase‐related GO term enrichment agreed with the distinct upregulation of phosphatidylinositol signaling system suggested in the KEGG enrichment. Furthermore, as part of phosphatidylinositol signaling system, *pik3c* exhibited an upregulated expression level according to the DE results. The gene encodes a key regulator of the PI3K‐PKB pathway, which has been proven vital to energy metabolism, cell proliferation and growth, as well as various aspects of muscle regulation (Glass 2010; Hemmings and Restuccia 2015). Thus, it is not surprising that the pathway is closely related to HIIT response and in particular muscle growth (Gibala 2009). Additionally, PI3K‐PKB pathway also facilitates fatty acid metabolism in liver and adipose tissue upon HIIT (Marcinko et al. 2015). As such, our results not only confirmed previous reports, but also demonstrate that hair follicles are capable of reflecting such responses. Moreover, the overall activation of kinase signaling seen in the hair follicles might also contribute to stress‐responsive signaling pathways, such as MAPK signaling, another key signal transduction cascade critical to HIIT responses (Lu et al. 2015).

Potentially linked by the aforementioned modified cell signal transduction, a range of metabolic and cell growth/proliferation themes were also enriched in the GS analysis, possibly contributing to the HIIT‐responsive physiological adaptations, especially in the context of muscle regulation. Consistent with the signs of PI3K‐PKB pathway activation, a selection of metabolism‐related downstream processes were enriched in our GS analysis. Shown as distinct upregulations, GO BP terms such as Monocarboxylic acid metabolic process, Cellular protein metabolic process, and Translation suggested activation of the protein metabolism. Additionally, distinctly upregulated KEGG pathways, such as Ribosome and proteasome are further indicative of the involvement of HIIT‐responsive protein metabolism in scalp hair follicles. These results are in agreement with the findings of previous reports showing protein metabolism is part of the muscle HIIT responses (Gibala 2009; Pugh et al. 2015). In addition to protein metabolism, the KEGG pathway analysis enriched several pathways related to energy metabolism, including upregulated Oxidative phosphorylation. The overrepresentation of the oxidative phosphorylation pathway in the post‐HIIT group was consistent with a previous study using a mouse model where HIIT‐responsive regulation of muscle respiration machinery was identified, including ATP synthesis, enzyme activity of CS, hydrogen peroxide production, as well as the complex V alpha‐unit level (Ramos‐Filho et al. 2015). Moreover, as depicted in Figure 6B, the elevated expression level of *cox* might contribute to such overrepresentation, and was also consistent with previous assessment in human skeletal muscle (Perry et al. 2008; Little et al. 2010). Beyond the cellular respiration pathway, the GS results further suggested that both carbohydrate and fatty acid metabolic themes to be HIIT responsive in human scalp hair follicles. For example, both RNA‐Seq and qPCR analysis showed an increased transcript level of *slc2a5*, which is responsible for glucose transport and facilitates carbohydrate metabolism (Barone et al. 2009; Le et al. 2013). The GS analysis also saw the enrichment of the PARR pathway as a distinct upregulation with elevated transcript level of *pparγ*, which could potentially be involved in fatty acid degradation in muscle and an indication of adipose tissue development upon HIIT, as suggested previously (Hoshino et al. 2013a; Marcinko et al. 2015). In addition to energy metabolism, scalp hair follicles might be able to provide transcriptomic information with regards to cell proliferation and growth, which are integral parts of the HIIT‐derived muscle adaptations (Gibala 2009; Pugh et al. 2015). Interestingly, the GO term themes related to cell proliferation exhibited enrichment in both directions, with distinctly upregulated terms, such as Regulation of cell proliferation, and downregulated term like Cell cycle. These results suggest that the HIIT‐responsive cell proliferation regulation in hair follicle is a highly complex process. Moreover, it appears that scalp hair follicles reflect HIIT‐associated muscle adaptations through the distinct upregulation of the KEGG pathway Smooth muscle contraction (Fig. 6A). It is worth noting that a potential activation of extracellular signal–regulated kinases (ERK) signaling (part of the MAPK cascades) was featured in such enrichment, confirming the involvement of MAPK signaling cascades in HIIT response. Taken together, our results show that scalp hair follicles are capable of providing transcriptomic responses upon HIIT in the context of energy metabolism and muscle adaptations.

The potential activation of stress signaling pathways, such as PI3K‐PKB and ERK in scalp hair follicles suggested scalp hair follicles can be an accurate surrogate of HIIT‐related stress responses (Smyth 2005; Altman and Rathmell 2012). For example, activation of these pathways may lead to prosurvival responses. Indeed, the DE results exhibited increased transcript levels of prosurvival genes including *b‐cell lymphoma‐extra large* (*bcl‐xl*) and *proto‐oncogene serine/threonine‐protein kinase* (*pim1*), indicating HIIT‐responsive antiapoptotic adaptations. Such observation has been previously reported in post‐HIIT rat hearts (Lu et al. 2015). Given its roles in regulating apoptosis (Gibala et al. 2012; Lu et al. 2015), the aforementioned activation of PPAR pathway might also contribute to the HIIT‐induced prosurvival responses. Consistently, both qPCR and DE results suggested an upregulated expression of the miRNA *let‐7g*, which was previously demonstrated to be responsible for inhibiting apoptosis introduced by oxidized low‐density lipoprotein (Zhang et al. 2013). Moreover, additional stress‐responsive pathways were revealed in the KEGG pathway analysis upon HIIT. For example, JAK‐STAT pathway was identified as a distinctly downregulated signaling pathway. While also closely related to cell stress responses and as a prosurvival signaling pathway (Rawlings et al. 2004), the current results suggested that JAK‐STAT pathway might be inhibited. As shown in Figure 5A, the increase in the transcript levels of *pi3k* and antiapoptotic genes confirmed that pathways other than JAK‐STAT might be responsible for the potential activation of the prosurvival machineries. The DE results suggested that the potential inhibition of the pathway could be mediated by the upstream cytokines and hormones, as well as the core transcription factor STAT. Such inhibition of JAK‐STAT pathway might be in favor of facilitating other aspects of HIIT responses in hair follicles. In fact, it appeared that the inhibited cytokine was featured in enriched pathways beyond JAK‐STAT, with Cytokine‐cytokine interaction enriched as distinctly downregulated KEGG pathway. These results were consistent with multiple previous studies in which only moderate IRs or no substantial activation of anti‐inflammatory machinery were observed upon HIIT (Zwetsloot et al. 2014; Cullen et al. 2016; Kaspar et al. 2016). Moreover, the distinctly downregulated KEGG pathway Steroid hormone biosynthesis might be the linked to the observed inhibition of JAK‐STAT associated hormone expression, as previously reported (Gupta and Mayer 2013). The current observations provided convincing evidence that scalp hair follicles are strikingly capable of reflecting such HIIT adaptations.

Accordingly, this study explored the transcriptomic adaptations when using human scalp hair follicles as a minimally invasive tool for the characterization HIIT. On the basis of RNA‐Seq transcriptome profiling, we used a comprehensive bioinformatics strategy for both DE and GS analyses to examine the capacity of the hair follicles to exhibit well‐characterized HIIT responsive molecular events on a transcriptome level. Comparing between pre and posttraining subjects, the present results showed that hair follicle transcriptome indeed exhibited enrichment of HIIT‐associated biomolecular events, such as GPCR‐and kinase‐mediated signal transduction, energy metabolism and muscle adaptations, cell stress responses and survival, as well as IR. These results are thematically consistent with the transcriptome response in the muscle, reported by Nishida et al. (2010).

Our DE analysis also showed adjusted expression patterns for the key genes linked to the aforementioned pathways. Additionally, the increased level of the miRNA *miR‐99a* also exhibited a potential hair follicle‐specific response upon HIIT, given the critical role of the *miR‐99a* in regulating keratinocyte differentiation (Lerman et al. 2011). These findings indicate that the hair follicle‐specific processes were responsive to HIIT. Based the results, the current limitations of using scalp hair follicle as a transcriptomic biomarker mainly resides in the varied visibility and availability of the hair shaft for sample acquisition. Building upon the findings of this study, future assessment can be focused on the hair follicle transcriptome sensitivity to the intensity and duration of the training bouts. By adding more biological replicates, follow‐up studies can also implement higher level bioinformatics techniques such as machine learning gene selection and classification for a more intelligent biomarker discovery using high‐throughput datasets.

For the first time, our study presented promising results that suggested transcriptomic profiling of human scalp hair follicles might have the potential to serve as both a research tool and transcriptomic biomarker discovery source for HIIT.

## Conflict of Interest

Opinions expressed or implied in this publication are those of the authors and do not represent the views or policy of the Department of National Defence or the Canadian Armed Forces. The authors declare that they have no competing interests.

## Supporting information




**Table S1.** (A) Complete list for protein coding gene differential expression results, showing Gene ID, Gene Name, Chromosome, logFC (log_2_ transformed fold change), Overall average expression level, *t* value (moderated *t*‐statistics), raw *P* value, and FDR adjusted *P* value. (B) Complete list for miRNA differential expression results, showing Gene ID, Gene Name, Chromosome, logFC (log_2_ transformed fold change), Overall average expression level, *t* value (moderated *t*‐statistics), raw *P* value, and FDR adjusted *P* value.
